# Editorial: Impact on Embryonic Development of Chromatin Remodeling Alterations

**DOI:** 10.3389/fcell.2021.744665

**Published:** 2021-08-12

**Authors:** Cristina Gervasini, Mario Garcia-Dominguez, Valentina Massa

**Affiliations:** ^1^Department of Health Sciences, Università degli Studi di Milano, Milan, Italy; ^2^“Aldo Ravelli” Center for Neurotechnology and Experimental Brain Therapeutics, Università degli Studi di Milano, Milan, Italy; ^3^Andalusian Center for Molecular Biology and Regenerative Medicine-CABIMER, Consejo Superior de Investigaciones Científicas (CSIC)-Universidad de Sevilla-Universidad Pablo de Olavide, Seville, Spain

**Keywords:** chromatin, epigenetic, embryo, development, molecular mechanisms

Despite the growing evidence on impact of chromatin remodeling alterations in several genetic disorders, the comprehension of embryonic consequences remains a scientific challenge.

The correct embryonic patterning is finely regulated and epigenetic changes are crucial to modulate the chromatin accessibility and gene expression.

The numerous inborn genetic disorders caused by mutations in genes coding chromatin players prove that the consequences of chromatin alterations seriously affect embryonic development.

For instance, since the idea of a unique group of syndromes named “Mendelian disorders of the epigenetic machinery” was proposed by Bjornsson in 2015 the number of recognized chromatinopathies has grown from 44 to 82 in 2019 (Bjornsson, 2015; Fahrner and Bjornsson, 2019).

In this special issue “Impact on Embryonic Development of Chromatin Remodeling Alterations” papers elucidating the mechanisms linking the chromatin-gene alteration and its effect on embryonic development were published.

The Brief Research Report titled “Olfactory Malformations in Mendelian Disorders of the Epigenetic Machinery” by Aleo et al. describes the olfactory malformation in a heterogeneous group of MDEM (mendelian disorder of epigenetic machinery). Over 30% of analyzed samples present an olfactory malformation suggesting a link between the chromatin remodeling alteration to the embryonic development of the olfactory bulbs.

Two comprehensive reviews by Parodi et al. and Lettieri et al. present data on human pathologies. in “Chromatin Imbalance as the Vertex Between Fetal Valproate Syndrome and Chromatinopathies” reported literature data support a molecular relationship between fetal valproate spectrum disorders (FVSD) and specific chromatinopathies, as if these conditions with obvious divergent etiologies, result from a similarly altered development. In “Semaphorin Regulation by the Chromatin Remodeler CHD7: An Emerging Genetic Interaction Shaping Neural Cells and Neural Crest in Development and Cancer” CHARGE syndrome- causative gene, CHD7 and its molecular interaction with semaphorins are extensively described. Their function at cellular level is of paramount importance in embryonic development and in tumor pathogenesis.

In the original research papers by Deng et al., Luo et al., Ketharnathan et al. and Zhang et al. using different animal models (goat, pig, zebrafish, and mouse) the role of five chromatin-genes (YTHDF2, SIN3A, Stag1, Stag2, and Jmjd3) is investigated. Interestingly, the presented data highlight the crucial role for such proteins across evolution and in different time-point of embryonic development and tissues.

The Hypothesis and Theory paper by Sui and Peng titled “A Mechanism Leading to Changes in Copy Number Variations Affected by Transcriptional Level Might Be Involved in Evolution, Embryonic Development, Senescence, and Oncogenesis Mediated by Retrotransposons” propose a role for CNVs and transcriptional regulation in fundamental cell division physiological processes and a possible contribution to developmental abnormalities, neurodegeneration and oncogenesis. In this suggested theory the information stored in the genome and its epigenetic regulation should be intended as bidirectional.

In conclusion, in line with the broad scope of Frontiers in Cell and Developmental Biology and the section on developmental epigenetics, this special issue addresses fundamental cellular and molecular mechanisms underlying the relevance of epigenetic control in directing early events shaping life.

## Author Contributions

CG, MG-D, and VM conceived and wrote the manuscript. All authors contributed to the article and approved the submitted version.

## Conflict of Interest

The authors declare that the research was conducted in the absence of any commercial or financial relationships that could be construed as a potential conflict of interest.

## Publisher's Note

All claims expressed in this article are solely those of the authors and do not necessarily represent those of their affiliated organizations, or those of the publisher, the editors and the reviewers. Any product that may be evaluated in this article, or claim that may be made by its manufacturer, is not guaranteed or endorsed by the publisher.
